# Targeting DNA-dependent protein kinase sensitizes hepatocellular carcinoma cells to proton beam irradiation through apoptosis induction

**DOI:** 10.1371/journal.pone.0218049

**Published:** 2019-06-13

**Authors:** Changhoon Choi, Arang Son, Ga-Haeng Lee, Sung-Won Shin, Sohee Park, Sang Hee Ahn, Yoonsun Chung, Jeong Il Yu, Hee Chul Park

**Affiliations:** 1 Department of Radiation Oncology, Samsung Medical Center, Seoul, South Korea; 2 Sungkyunkwan University School of Medicine, Seoul, South Korea; 3 Department of Nuclear Engineering, Hanyang University, Seoul, South Korea; Chung Shan Medical University, TAIWAN

## Abstract

Recent studies have highlighted the implications of genetic variations in the relative biological effectiveness (RBE) of proton beam irradiation over conventional X-ray irradiation. Proton beam radiotherapy is a reasonable radiotherapy option for hepatocellular carcinoma (HCC), but the impact of genetic difference on the HCC RBE remains unknown. Here, we determined proton RBE in human HCC cells by exposing them to various doses of either 6-MV X-rays or 230-MeV proton beams. Clonogenic survival assay revealed variable radiosensitivity of human HCC cell lines with survival fraction at 2 Gy ranging from 0.38 to 0.83 and variable proton RBEs with 37% survival fraction ranging from 1.00 to 1.48. HCC cells appeared more sensitive to proton irradiation than X-rays, with more persistent activation of DNA damage repair proteins over time. Depletion of a DNA damage repair gene, DNA-PKcs, by siRNA dramatically increased the sensitivity of HCC cells to proton beams with a decrease in colony survival and an increase in apoptosis. Our findings suggest that there are large variations in proton RBE in HCC cells despite the use of a constant RBE of 1.1 in the clinic and targeting DNA-PKcs in combination with proton beam therapy may be a promising regimen for treating HCC.

## Introduction

Radiation therapy is recognized as effective alternative option for treating liver cancers such as hepatocellular carcinoma (HCC) [1–3]. Advanced radiation techniques such as stereotactic body radiotherapy improve clinical outcomes in patients with unresectable primary HCC. Accumulating evidence indicates that charged particle beam therapy such as protons and carbon ions is promising for HCC, as these techniques lead to better tumor control and minimal toxicity in normal tissues due to dosimetric advantages over conventional radiotherapy [4–9].

Proton beam therapy is currently performed based on the proton relative biological effectiveness (RBE) of 1.1 relative to photons [10,11]. A generic RBE value of 1.1 represents an averaged value of estimates from numerous experiments *in vitro* and *in vivo* [10] and there is no clinical evidence that this value is incorrect, even though this generic value ignores all the possible variations [12]. The RBE depends on a variety of factors including dose, linear energy transfer (LET), tissue type, and biological end points, among others [10]. Recent biological studies have demonstrated that the RBE is also affected by differences in genetic background, indicating the need for suitable biomarkers that predict response to proton therapy [11,13].

The repair capacity or efficiency of lethal DNA damages such as double strand breaks (DSBs) is central to determining the cellular response to particle therapy as well as conventional radiation therapy. Selective inhibition of DNA DSB repair pathways, non-homologous end joining (NHEJ) and homologous recombination (HR), elicits differential responses to proton versus photon irradiation [14]; targeting the HR pathway or the Fanconi anemia (FA) pathway strongly sensitizes lung adenocarcinoma and ovarian carcinoma cells to proton irradiation [14,15]. Pharmacological inhibition or gene silencing of DNA-PKcs, a key player in the NHEJ pathway, sensitizes cancer cells to ionizing radiation including X-ray and carbon ion beams [16–20]. In HCC, the clinical benefit of DNA-PKcs inhibition has been actively exploited because increased expression and activity of DNA-PKcs is an independent biomarker for poor survival in HCC [21–23].

Predictive biomarkers or combination strategies for proton therapy have been identified [15,24–26]. In this study, we aimed to compare for the first time the biological effectiveness of protons relative to X-rays in HCC cell lines using therapeutic radiation therapy machines and screen for genetic factors that could potentially affect proton RBE. We found large variations in proton RBE values and DNA damage signaling among HCC cell lines. Our findings that DNA-PK inhibition selectively enhanced proton sensitivity suggest that a comprehensive understanding of genetic factor(s) contributing to RBE may be necessary to improve proton therapy efficacy.

## Materials and methods

### Cell culture

Eight human hepatocellular carcinoma cell lines, Hep3B, Huh7, HepG2, PLC/PRF/5, SK-HEP-1, SNU-182, SNU-387, and SNU-449, were purchased from the Korean Cell Line Bank (Seoul National University, Seoul, Korea), cultured in Dulbecco’s modified Eagle medium (DMEM), and supplemented with 10% fetal bovine serum (FBS) and 25 mM HEPES (Gibco, Carlsbad, CA, USA). Human salivary gland (HSG) cell line was a generous gift from Dr. Eunho Kim at Korea Institute of Radiological & Medical Sciences, South Korea, with permission of Dr. Yoshiya Furusawa at National Institute of Radiological Sciences, Japan. The HSG cells were cultured in Eagle’s minimum essential medium supplemented with 10% FBS and 1x antibiotic-antimycotic. Cultures were maintained in a humidified atmosphere of 95% air/5% CO_2_ at 37°C.

### Reagents and antibodies

Anti-phospho-H2AX (Ser139, 07–164) antibodies were purchased from Millipore. Anti-phospho-CHK2 (Thr68, #2197), cleaved PARP (#9541), cleaved caspase-3 (#9661), phospho-p38 MAPK (Thr180/Tyr182, #4511), and DNA-PKcs (#4602) antibodies were purchased from Cell Signaling Technology (Danvers, MA, USA). Anti-β-actin antibodies were purchased from Sigma Aldrich (St. Louis, MO, USA). Anti-phospho-DNA-PKcs (S2016, ab18192) and phospho-ATM (S1981, 200-301-400) antibodies were purchased from Millipore (Billerica, MA, USA) and Rockland Immunochemicals (Limerick, PA, USA), respectively. Anti-BRCA1 (SC-6954) and Rad51 (SC-8349) antibodies were purchased from Santa Cruz Biotechnology (Santa Cruz, CA, USA). The siRNAs for gene knockdown experiments were purchased from Santa Cruz Biotechnology and RNAiMax (13778–150) for transfection was purchased from Invitrogen (Carlsbad, CA, USA).

### Irradiation experiments

**Fig 1:**
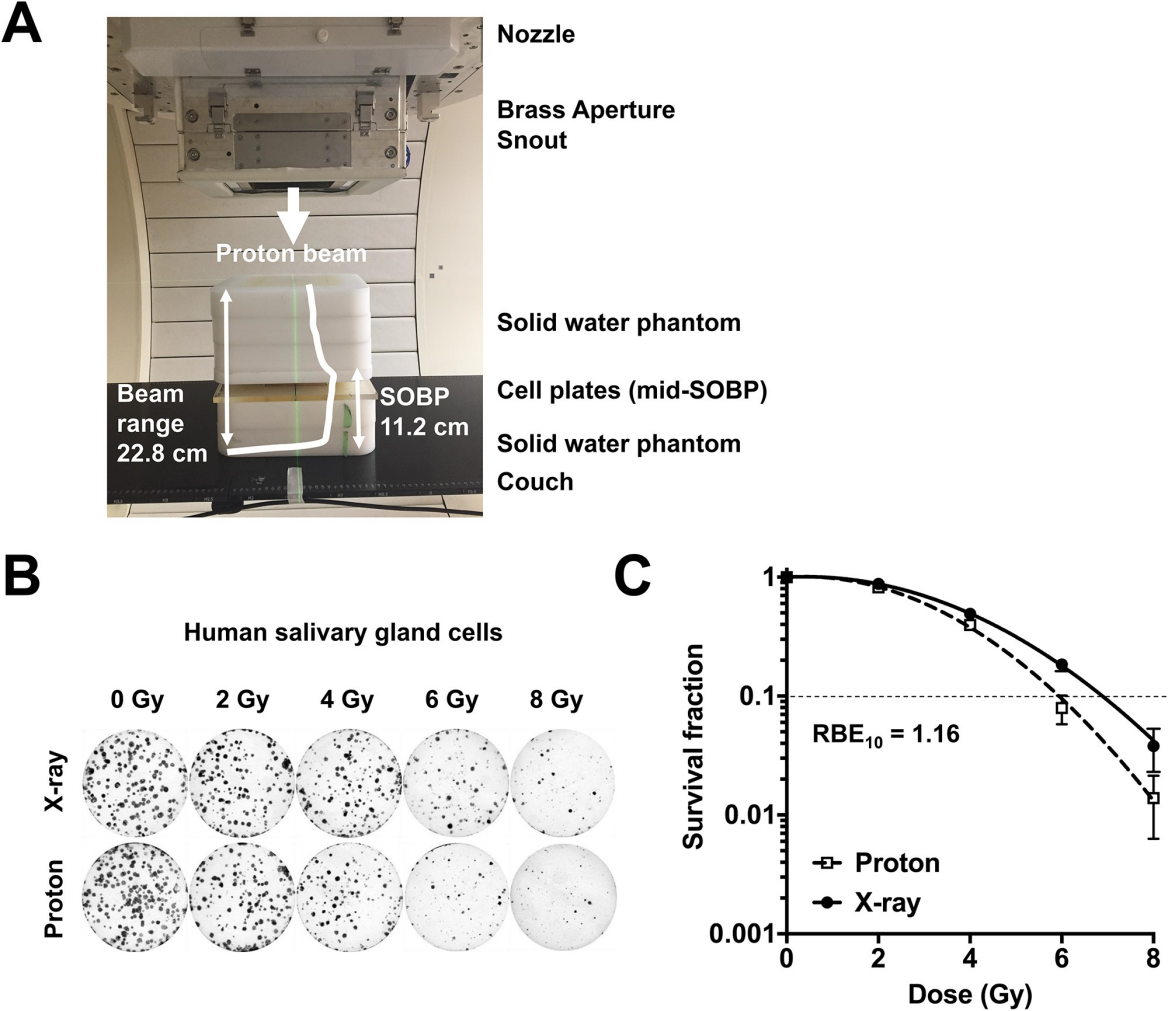
Estimation of *in vitro* RBE at Samsung Proton Therapy Center. (**A**) Proton irradiation set up. (**B**) Colony images of human salivary gland (HSG) cells formed after exposure to the indicated doses of X-rays or proton beams. (**C**) Dose-response curves of HSG cells. Dotted line indicates 10% survival. RBE was calculated as described in Materials and methods.

Proton beams were delivered by a proton therapy machine (Sumitomo Heavy Industries, Tokyo, Japan) at Samsung Proton Therapy Center in Seoul, Korea [27]. The cells were irradiated with 230 MeV proton beams using the wobbling method with a dose rate of 2.14 Gy/min. The proton beam range was 22.8 cm (distal 90%) and the spread-out Bragg peak (SOBP) width was 11.2 cm. The cells were positioned in the middle of the spread-out Bragg peak (SOBP) within a field collimated by 18 × 12-cm Brass blocks. The proton irradiation setup was presented in Fig 1A. The absolute proton beam dose was verified to 1% accuracy according to TRS-398 for proton therapy. X-ray irradiation was performed with 6-MV photons with a dose rate of 3.96 Gy/min using a linear accelerator Varian Clinac 6EX (Varian Medical Systems, Palo Alto, CA, USA). The cells were placed under a 2-cm thick solid water phantom with the source surface distance of 100 cm and the field size of 30 cm × 30 cm. The absolute X-ray dose was verified according to TG-51 with Gafchromic film to 1% accuracy.

### Clonogenic survival assay

The cell plates were exposed to X-ray or proton beams with physical doses of 0, 2, 4, 6 and 8 Gy and incubated for 7–14 days. After staining with 0.5% crystal violet (Sigma-Aldrich), colonies that contained more than 50 cells were considered viable and counted. To obtain cell survival data, the survival fraction (SF) was calculated as a ratio of the plating efficiency (ratio of the number of surviving colonies to the number of plated cells) of the irradiated cells to the plating efficiency of unirradiated cells. The survival curves were fitted with a linear-quadratic model [SF = exp(-αD-βD^2^); SF, survival fraction; D, absorbed dose] and analyzed using GraphPad Prism 7.02 (GraphPad Software, La Jolla, CA, USA) or an in-house RBE analysis program [28]. Proton RBE_50_, RBE_37_ and RBE_10_ were defined as the ratio of the physical dose of X-ray to the dose of proton that yielded the same survival fractions of 50%, 37% and 10%, respectively. Dose enhancement factor (DEF) by siRNA treatments was calculated as the ratio of the physical doses at 50% survival.

### Transfection

Transfection was performed using Lipofectamine RNAiMax transfection reagent (Invitrogen, Carlsbad, CA, USA) according to the manufacturer’s instructions. Briefly, the cells were seeded to 70% confluence in 10-cm dishes a day before transfection. The control siRNAs (sc-37007) and DNA-PKcs siRNAs (sc-35200) were purchased from Santa Cruz Biotechnology (Santa Cruz, CA, USA) and diluted to 10 nM of the final concentration in Opti-MEM and incubated with lipofectamine RNAiMax at a 1:1 ratio for 15 min. The siRNA-lipofectamine complex was then transferred to cell dishes.

### Western blot analysis

The cell lysates were prepared in a lysis buffer (20 mM Tris (pH 8.0), 137 mM NaCl, 10% glycerol, 1% Nonidet P-40, 10 mM EDTA, 100 mM NaF, 1 mM phenylmethylsulfonyl fluoride and 10 mg/ml leupeptin). After centrifugation at 13,000 rpm for 15 min, the protein concentration in each lysate was determined using Bio-Rad protein assay reagent (Bio-Rad, Richmond, CA, USA) according to the manufacturer’s protocol. Equal amounts of proteins were loaded and separated by SDS-PAGE. After transfer to nitrocellulose membranes (Bio-Rad), blots were blocked overnight with 5% skim milk in PBS at 4°C and probed with primary antibody overnight. Protein bands were detected with Amersham enhanced chemiluminescence detection reagents (GE healthcare, Piscataway, NJ, USA).

### Cell cycle analysis

After 2 h, 24 h and 72 h of X-ray or proton irradiation, the cells were harvested and fixed with pre-chilled 70% ethanol. For propidium iodide (PI) staining, the cells were incubated with 1 mg/ml RNase and 50 μg/ml PI in the dark for 30 min at 37 °C. Cell cycle distribution was analysed by flow cytometry using a BD FACSVerse flow cytometer (BD Biosciences, New Jersey, USA) and a BD FACSuit software.

### Apoptosis analysis

After 72 h of X-ray or proton irradiation, the cells were incubated with annexin V-FITC (BD Pharmingen, San Diego, CA, USA) and 2 μg/ml PI in annexin V binding buffer (10 mM HEPES, pH 7.4, 140 mM NaCl, 2.5 mM CaCl_2_) in the dark for 15 min at 37°C. The apoptotic cell populations were analysed using a BD FACSVerse flow cytometer and a BD FACSuit software.

### Real-time quantitative reverse transcription-polymerase chain reaction (qRT-PCR)

SNU-449 cells were plated at 1 × 10^5^ cells/100 mm dish, and 72 h after irradiation, total RNA was extracted using TRIzol (Invitrogen, Carlsbad, CA, USA) according to the manufacturer’s instructions. The gene expression was assessed by performing real-time qRT-PCR using One-Step TB Green PrimeScript RT-PCR Kit II (Takara Bio, Shiga, Japan) and a QuantStudio 3 Real-Time PCR System (Applied Biosystems, Foster City, CA, USA). Relative gene expression was calculated with the 2^-ΔΔCt^ method and GAPDH was used as an internal control. The primer sets (Bioneer, Daejeon, Korea) were as follows: DNA-PKcs forward: 5’-TGCAGTCTTCAGTGGATAATACC-3’, DNA-PKcs reverse: 5’-CACTGCCATTTTAGTTTCGAGAG-3’; Bcl-2 forward: 5’-GTCCAAGAATGCAAAGCACAT-3’, Bcl-2 reverse: 5’-CTCTGCGACAGCTTATAATGGA-3’; Bak forward: 5’-ATGGTCACCTTACCTCTGCAA-3’, Bak reverse: 5’-TCATAGCGTCGGTTGATGTCG-3’; GAPDH forward: 5’-ACAGTCAGCCGCATCTTCTT-3’, GAPDH reverse: 5’-CGCCCAATACGACCAAATCC-3’.

### Statistical analysis

Data are presented as the mean ± standard deviation (SD). Statistical analysis was performed using GraphPad Prism 7.02. The statistical significance of differences between experimental groups was calculated with an unpaired, two-tailed Student’s t-test. The values of p < 0.05 were considered statistically significant.

## Results

### HCC cell lines have varying sensitivity to X-ray and proton irradiation

Before measuring radiosensitivity in HCC cells, we first determined proton RBE in HSG cells, which were used as a refence cell line for RBE comparison at multiple proton therapy facilities in japan, as a biological quality assurance process [29]. A clonogenic survival assay showed that proton irradiation was slightly more effective than X-rays (Fig 1B) and the dose-response curves showed that proton RBE at 10% survival was estimated as 1.16 (Fig 1C). This was very close to the RBEs from other proton therapy facilities using the same biological system.

**Fig 2:**
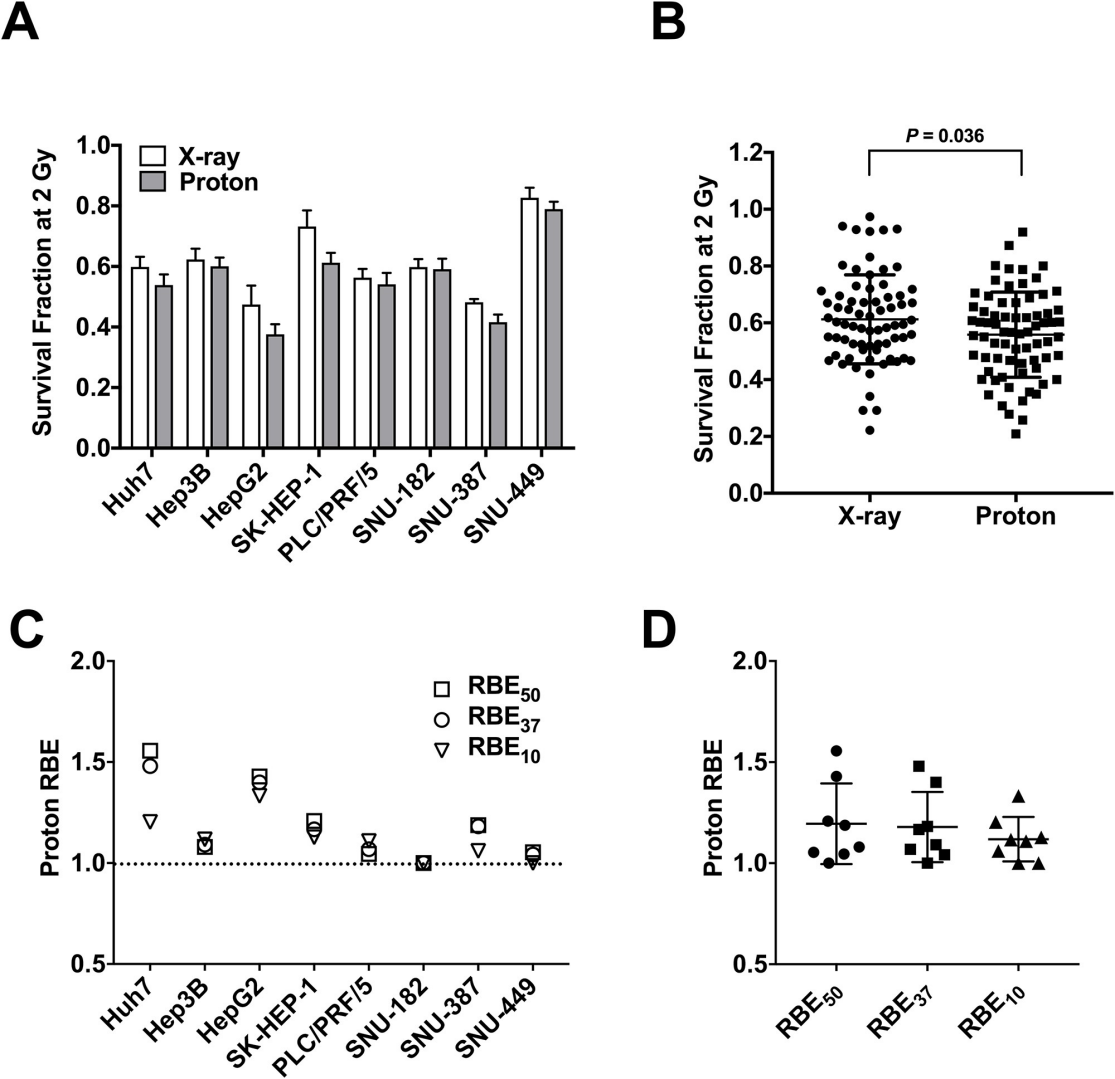
Radiation sensitivity and proton RBEs differ largely in HCC cell lines. (**A**) Bar graph shows variability of radiosensitivity of eight HCC cell lines. Radiosensitivity was compared as survival fractions at 2 Gy (SF2) of X-ray and proton irradiation. Data represent mean ± SD from three independent experiments. (**B**) Column scatter plot shows that protons were more effective than X-rays in HCC cells (p < 0.05). (**C**) Comparison of RBEs of eight HCC lines at survival fractions of 50% (RBE_50_), 37% (RBE_37_), and 10% (RBE_10_). The dashed line indicates RBE of 1.0. (**D**) Summary of proton RBEs in HCC cell lines. The box-and-whisker plot indicates averaged RBE values of approximately 1.1, regardless of biological end points.

Based on previous reports on large variations of proton RBE in lung cancer [15] and head and neck cancer cell lines [30], we attempted to determine proton RBE in liver cancer cell lines. We irradiated eight human hepatocellular carcinoma cell lines with X-rays or protons with the same physical doses ranging from 0 to 8 Gy. Dose-response curves revealed that most HCC cell lines had varying sensitivity to X-ray and proton irradiation (Fig 2A) and they were more sensitive to proton beam irradiation than X-ray irradiation (Fig 2B, p < 0.05). When survival fraction at 2 Gy (SF2) was compared, HepG2 cells (0.38 ± 0.033 and 0.48 ± 0.062) were the most sensitive to both irradiations and SNU-449 cells (0.79 ± 0.025 and 0.83 ± 0.033) were the most resistant (Fig 2A).

Next, proton RBE in HCC cell lines was determined. The RBEs of Huh7, HepG2, and SK-HEP-1 were higher than 1.2, regardless of the survival fractions (Fig 2C). The RBEs of Hep3B, PLC/PRF/5 and SNU-387 were around 1.1, which is close to the fixed RBE value being used clinically. Interestingly, SNU-182 and SNU-449 cells had an RBE of 1.0, which indicates little difference in cell killing effect between X-rays and protons (Fig 2C). From a data set using eight human HCC cell lines, the averaged RBE_50_, RBE_37_ and RBE_10_ were 1.20, 1.18 and 1.12, respectively (Fig 2D). Dose response curves of eight HCC cell lines are presented in S1 Fig and parameters of survival assay of HCC cell lines are listed in Table 1.

**Table 1:** Parameters of survival assay of HCC cells irradiated with proton beams or X-rays.

Cell lines	SF2-proton	SF2-X-ray	RBE_50_	RBE_37_	RBE_10_
Huh7	0.54 ± 0.035	0.60 ± 0.033	1.56	1.48	1.2
Hep3B	0.60 ± 0.029	0.62 ± 0.036	1.08	1.09	1.12
HepG2	0.38 ± 0.033	0.48 ± 0.062	1.43	1.40	1.33
SK-HEP-1	0.61 ± 0.032	0.73 ± 0.053	1.21	1.17	1.13
PLC/PRF/5	0.54 ± 0.037	0.56 ± 0.029	1.05	1.07	1.11
SNU-182	0.59 ± 0.035	0.60 ± 0.026	1.00	1.00	1.00
SNU-387	0.42 ± 0.025	0.48 ± 0.011	1.19	1.18	1.06
SNU-449	0.79 ± 0.025	0.83 ± 0.033	1.05	1.04	1.00

Data are presented as mean ± S.E.M. from three independent experiments performed in triplicate. SF2: survival fractions at 2 Gy of proton beams or X-rays; RBE_50_, RBE_37_, RBE_10_: relative biological effectiveness defined as a ratio of the physical dose of X-ray and proton corresponding to 50%, 37% and 10% survival fractions.

### HCC cells show different DNA damage responses to X-ray and proton irradiation

**Fig 3:**
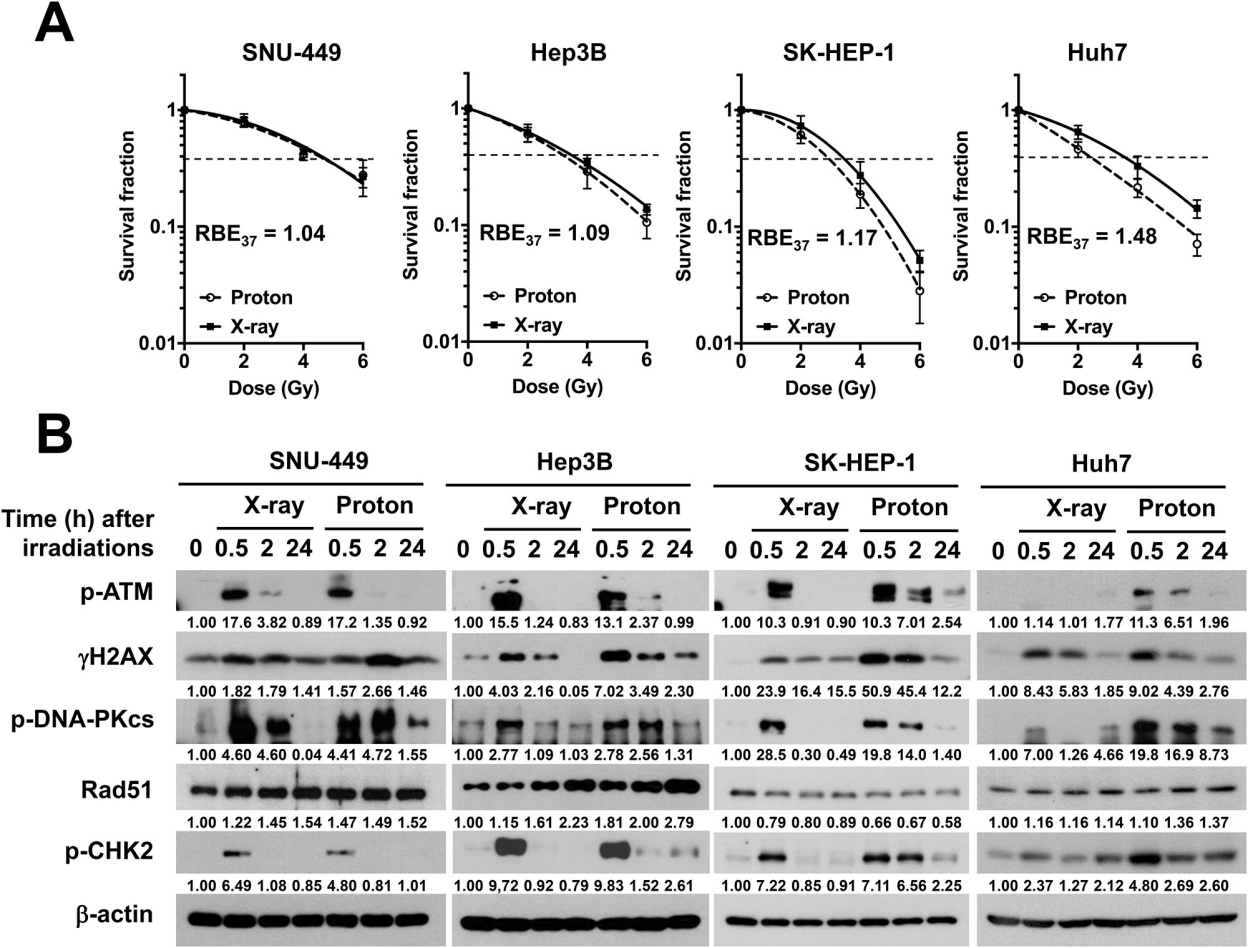
Cellular responses to X-rays and protons differ in HCC cell lines. (**A**) Dose-response curves of SNU-449, Hep3B, SK-HEP-1 and Huh7 against X-rays (solid curves) or protons (dashed curves). RBE_37_ of four HCC cell lines were compared (dotted lines). Data represent mean ± SD from three independent experiments and were fit in Graphpad Prism using a linear-quadratic model. (**B**) Time-dependent cellular responses to X-rays and protons in HCC cells. Each cell line was irradiated with 6 Gy of X-rays or protons and were harvested at the indicated times. Western blot analysis shows four HCC cell lines differs in DNA damage response signaling against X-rays or protons. β-actin was used as a loading control. p-ATM: phospho-ATM(S1981); γH2AX: phosphor-H2AX(Ser139); p-DNA-PKcs: phosphor-DNA-PKcs(S2016); p-CHK2: phospho-CHK2 (Thr68). Relative band intensity was quantified by densitometry using imageJ and the results normalized to β-actin are displayed under each blot.

Given that variable proton RBE may be due to DSB repair capacity, we investigated DNA damage response (DDR) signaling against X-ray or proton beam irradiation in four HCC cell lines, which had different RBE values. SNU-449, Hep3B, SK-HEP-1 and Huh7 cell lines had RBE_37_ of 1.04, 1.09, 1.17 and 1.48, respectively (Fig 3A). Time course analysis on the phosphorylation of the DDR markers such as ATM, H2AX, DNA-PKcs and CHK2 revealed there were slight difference in the DDR signaling between two groups (Fig 3B). The phosphorylation of DDR markers equally increased at 30 min post-irradiation with both X-rays and protons and returned to the basal level thereafter. Hep3B, SK-HEP-1 and Huh7, which had high RBE, showed prolonged activation of DDR following proton irradiation; the activated DDR signals remained more persistent in the proton-irradiated cells, as compared with the X-ray-irradiated cells. In contrast, SNU-449 cells, which had the lowest RBE, showed relatively few differences in the DDR signaling between X-rays and protons, compared to other cell lines.

### Depletion of DNA-PKcs increases proton sensitization in HCC cells

**Fig 4:**
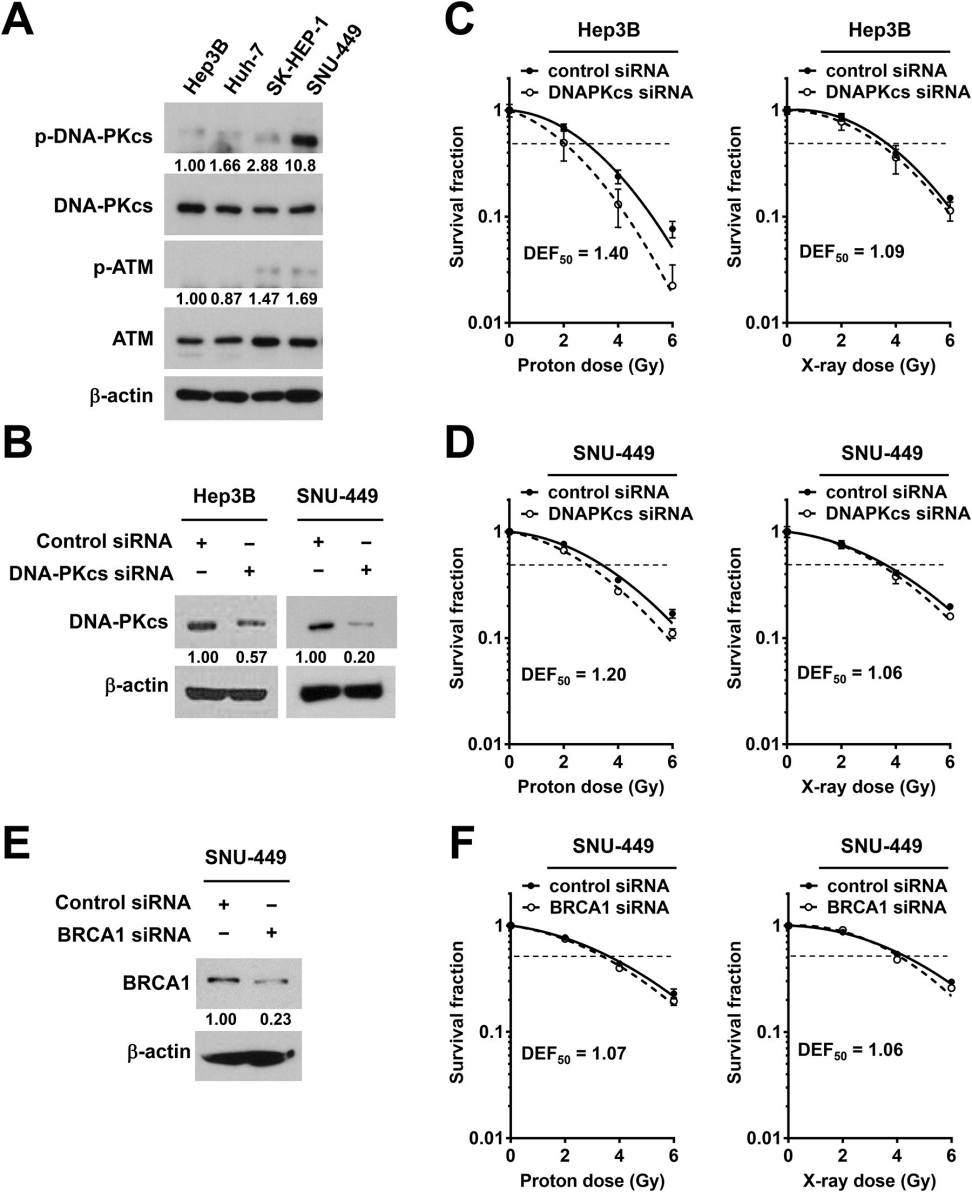
Depletion of DNA-PKcs sensitizes HCC cells to proton irradiation. (**A**) Detection of basal levels of DNA-PKcs and ATM, and their activated forms among four HCC cell lines. β-actin was used as a loading control. Intensity of phospho-DNA-PKcs and phosphor-ATM bands was normalized to total DNA-PKcs and ATM, respectively. (**B**) siRNA-mediated knockdown of DNA-PKcs in Hep3B and SNU-449. The cell lysates were prepared 24 h after transfection and were subjected to Western blot analysis. Relative expression of DNA-PKcs in knockdown cells versus control siRNA cells was shown. (**C** and **D**) Clonogenic survival assay showed that the depletion of DNA-PKcs by siRNA enhanced proton radiosensitization in Hep3B (**C**) and SNU-449 (**D**) cells. (**E**) Western blotting confirmed siRNA-mediated BRCA1 knockdown in SNU-449 cells. β-actin was used as a loading control. Relative expression of BRCA1 in knockdown cells versus control siRNA cells was shown. (**F**) Clonogenic assay showed that depletion of BRCA1 did not affect clonogenic survival of SNU-449 cells treated with protons or X-rays. All data represent mean ± SD and the survival curves were fitted to a linear-quadratic model using GraphPad Prism software. DEF_50_ was calculated as described in Materials and methods.

Among the DDR markers tested, it seemed that DNA-PKcs was more strongly and persistently activated by protons than by X-rays in four HCC cell lines (Fig 3B). To determine whether DNK-PKcs plays a certain role in response to proton irradiation in HCC cells, we tested the effect of DNK-PKcs depletion on proton sensitization. Among four HCC cell lines, the basal phosphorylation levels of DNA-PKcs but not those of ATM were clearly high in SNU-449, although the total levels of DNA-PKcs and ATM were not different (Fig 4A). The siRNA-mediated depletion of DNA-PKcs in Hep3B and SNU-449 cells was verified by Western blot analysis (Fig 4B). The clonogenic survival assay showed that DNA-PKcs siRNA greatly increased the sensitivity of Hep3B cells to protons; the DEF at 50% survival (DEF_50_) of 1.40 for protons in comparison with that of 1.09 for X-rays (Fig 4C). The depletion of DNK-PKcs by siRNA also sensitized SNU-449 cells to proton irradiation with the DEF_50_ of 1.2, compared to that of 1.06 for X-rays (Fig 4D). In contrast, when BRCA1, a DDR protein involved in HR pathway, was depleted in SNU-449 cells (Fig 4E), there was little effect on radiosensitization; the DEF_50_ of 1.07 and 1.06 for protons and X-rays, respectively (Fig 4F).

### Depletion of DNA-PKcs augments proton-induced apoptosis in HCC cells

**Fig 5:**
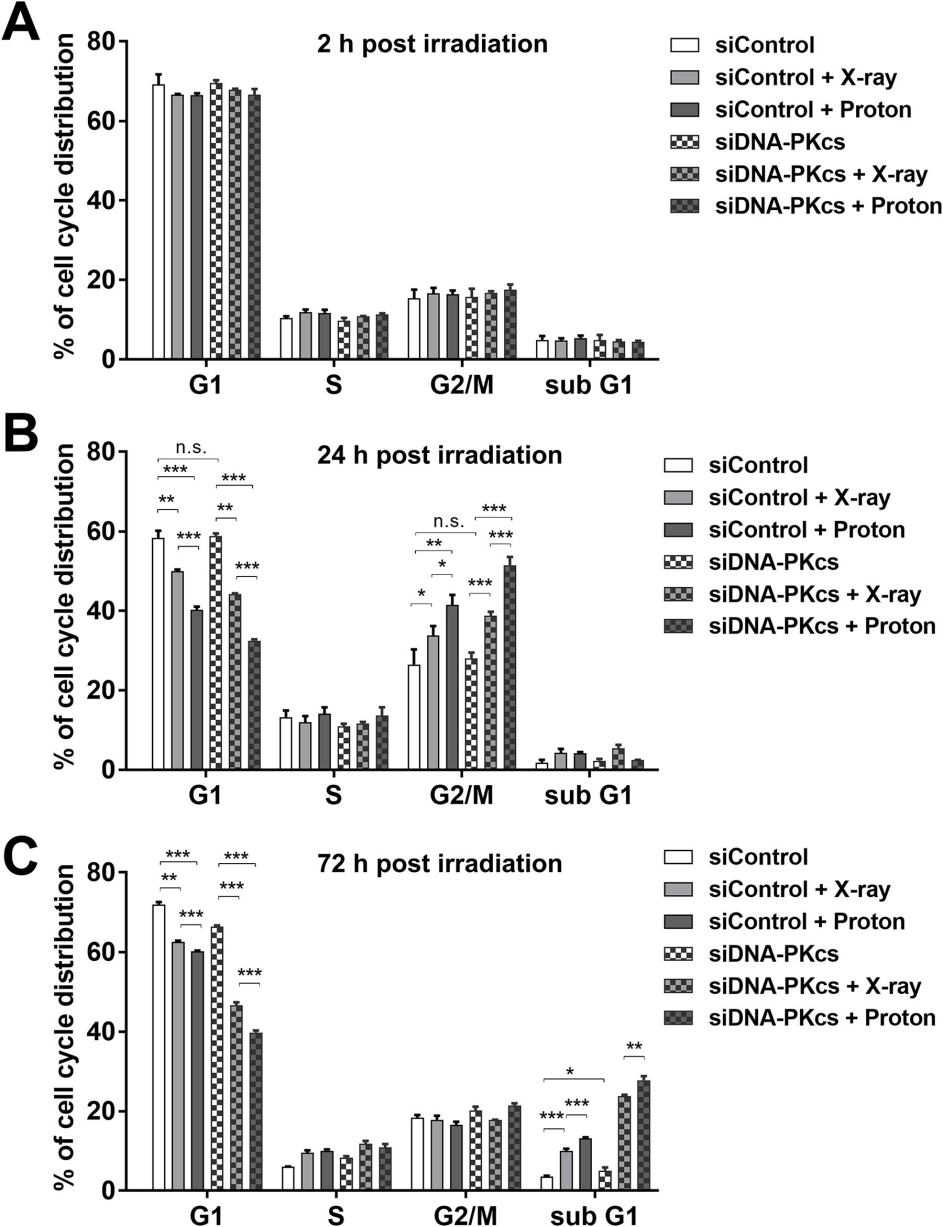
Depletion of DNA-PKcs alters cell cycle progression in SNU-449 cells after irradiation. The siRNA-transfected cells were irradiated with 6 Gy of X-rays or protons and were harvested at 2 h (**A**), 24 h (**B**) or 72 h (**C**). The cell cycle analysis was performed with flow cytometry and PI staining. Data represent mean ± SD from two independent experiments. *p < 0.05; **p < 0.01; ***p < 0.001; n.s.: not significant. The majority of cells remained at the G1 phase 2 h post-irradiation. G2/M phase cell populations increased 24 h post-irradiation and decreased thereafter concomitantly with an increase in subG1 phase. DNA-PKcs depletion further enhanced increased G2/M at 24 h and subG1 at 72 h by proton irradiation.

We determined the effect of DNA-PKcs depletion on the cell cycle progression after irradiation in SNU-449 cells. Flow cytometry analysis showed that both proton and X-ray irradiations did not affect cell cycle progression 2 h post-irradiation (Fig 5A) and they increased cell population at the G2/M phase with a concomitant decrease in the G1 phase cell population 24 h post-irradiation (Fig 5B); protons increased the G2/M phase cells more than did X-rays (p < 0.05). In the DNA-PKcs-deficient cells, the G2/M arrest was more clearly seen with protons than with X-rays (Fig 5B; p < 0.001). During re-entering the cell cycle 72 h post-irradiation, the G2/M populations dramatically decreased in all groups, while the subG1 populations increased only in the radiation-treated groups. The depletion of DNA-PKcs increased the subG1 population (p < 0.05) and further augmented the radiation-induced subG1 population (Fig 5C); protons accumulated the subG1 cells more than did X-rays (p < 0.01).

**Fig 6:**
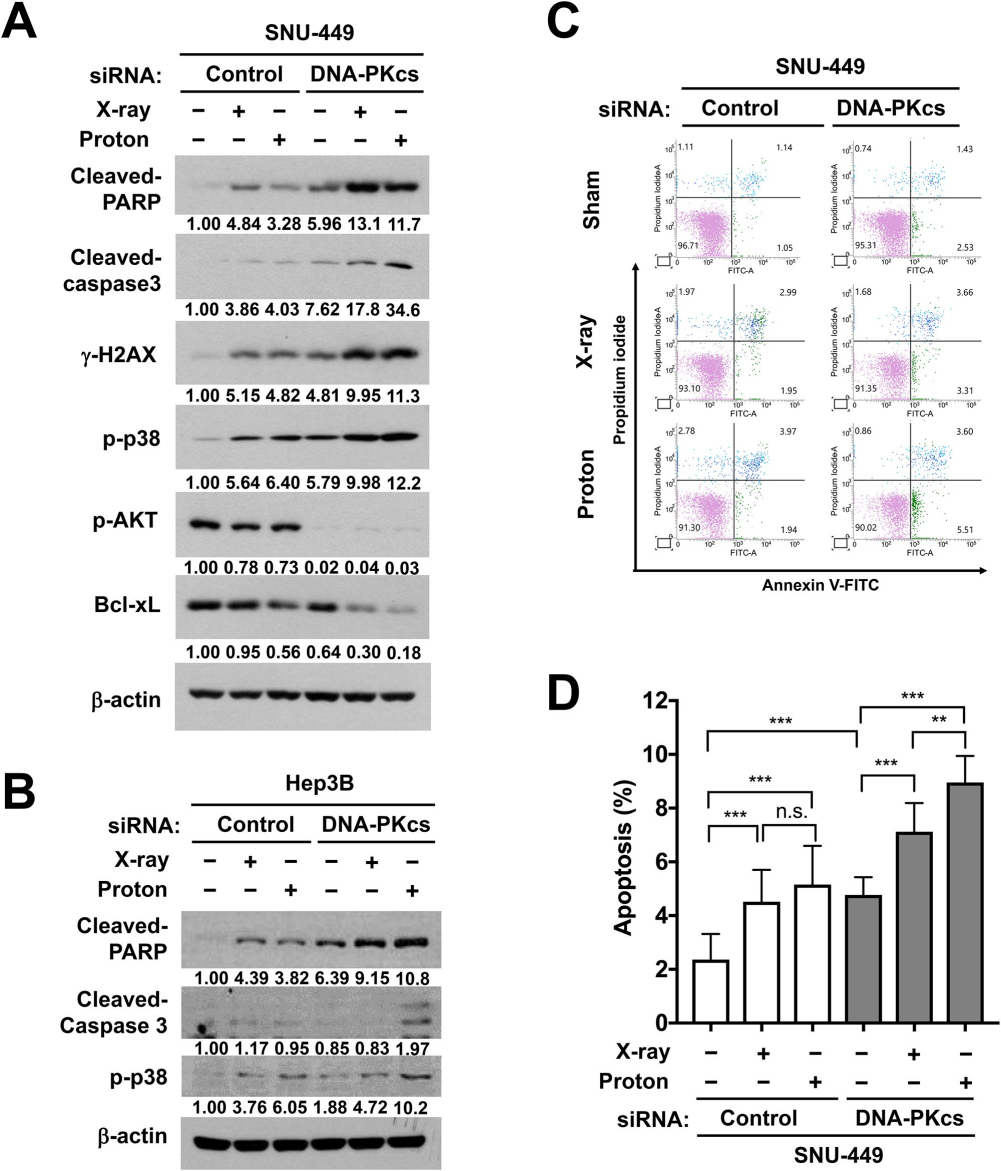
Depletion of DNA-PKcs increases proton sensitivity of HCC cells via induction of apoptosis. (**A**) Western blot shows induction of apoptotic markers by combination treatment with DNA-PKcs siRNA and protons in SNU449 cells. The siRNA-transfected cells were harvested 72 post-irradiation with 6 Gy of X-rays or protons. Expressions of cleaved-PARP, cleaved-caspase 3 and phopho-p38 were used as markers for apoptosis induction. Bcl-xL and phopho-AKT were used as pro-survival markers. β-actin was used as a loading control. Relative band intensity was normalized to β-actin. (**B**) DNA-PKcs depletion enhanced proton-induced apoptosis in Hep3B cells. The samples were prepared as described above. β-actin was used as a loading control. Relative band intensity was normalized to β-actin. (**C**) Flow cytometry histograms showing induction of apoptosis by combination with DNA-PK siRNA and protons. The siRNA-transfected SNU-449 cells were irradiated with 6 Gy of X-rays or protons and were harvested at 72 h. Apoptosis was assessed by flow cytometry with annexin V and PI co-staining. (**D**) Quantification of apoptosis shows that DNA-PK siRNA enhanced proton-induced apoptosis, compared to control-siRNA. Data represent mean ± SD from two independent experiments. **p < 0.01; ***p < 0.001; n.s.: not significant.

Next, we tested the combination effect of radiation and DNK-PKcs siRNA on apoptosis in HCC cells. A single treatment with DNA-PKcs siRNA or radiation increased the expression of the apoptosis markers, cleaved-PARP and caspase-3 in SNU-449 cells (Fig 6A). Combination treatment with DNA-PKcs siRNA and radiation enhanced the expression of apoptotic marker proteins, compared to the single treatment. Phosphorylation of p38, another apoptotic marker, was also increased by the combined treatment with radiation and DNA-PKcs siRNA. In contrast, the expression of Bcl-xL, an anti-apoptotic marker and phosphorylated AKT, a pro-survival marker, was greatly decreased in the combination treatment groups. The expression of γ-H2AX, a DSB marker, remained at 72 h post-irradiation and further increased by co-treatment with DNA-PKcs siRNA. Consistently, the depletion of DNK-PKcs enhanced proton-induced expression of apoptotic markers in Hep3B with increased expression of cleaved PARP and caspase 3 (Fig 6B). The induction of apoptosis was further confirmed by flow cytometry using annexin V/PI co-staining (Fig 6C and 6D). Both radiations increased apoptotic cell population in SNU-449 cells (p < 0.001) and DNA-PKcs depletion further enhanced radiation-induced apoptosis (p < 0.001); the DNA-PKcs depletion induced apoptosis more effectively in combination with protons than with X-rays (p < 0.01; Fig 6D). Furthermore, HepG2 cells were considered most sensitive at 2 Gy (Fig 2A) and they also showed a strong combination effect of DNA-PKcs depletion with protons compared to that with X-rays in terms of apoptotic cell death (p < 0.001; S2 Fig).

To determine how DNA-PKcs depletion affects apoptotic signaling in SNU-449 cells, the mRNA and protein expression levels of pro-apoptotic/anti-apoptotic markers were assessed by performing real-time qRT-PCR and western blot. DNA-PKcs siRNA efficiently suppressed mRNA expression of DNA-PKcs (S3 Fig). The combination with DNA-PKcs siRNA and protons increased the mRNA expression of Bak, a pro-apoptotic marker, but it did not regulate the mRNA expression of Bcl-2, an anti-apoptotic marker (S3 Fig). In contrast, the same combination increased the protein expression of Bak and concomitantly decreased the protein level of Bcl-2; the combination with DNA-PKcs siRNA and protons shifted the Bak/Bcl-2 ratio in favor of apoptosis (S3 Fig).

## Discussion

Cancer treatment with proton beams is increasing in popularity, with a growing number of proton therapy facilities worldwide. Proton therapy has been actively performed for liver cancer patients, especially in Asia, but the relative biological effects of proton beams over X-rays in liver cancer cells have not been measured yet. In this study, we aimed to determine the proton RBE in human HCC cell lines using clinical radiotherapy machines. Our proton RBE estimation using HSG cell line, one of the reference cell lines used for inter-institutional comparison, validated that the biological experiment setup of our proton facility was not different from that of other proton facilities [29,31,32]. Clonogenic survival assay using eight human HCC cell lines revealed large variation of radiation sensitivity in HCC cell lines. SNU-449 was the most resistant to both radiations. The RBE calculations revealed that the proton RBEs of HCC cells were also variable. Our data that indicated that seven out of eight HCC cell lines showed an RBE_50_ > 1.00 suggest higher biological effectiveness of protons over X-rays for HCC *in vitro*.

Our RBE screening in HCC cell lines showed that Huh7 cell line had the highest RBE estimates (RBE_50_ was 1.56). Huh7 cells have a functional defect in the FA pathway due to a nonsense mutation in the FANCC gene and the re-expression of FANCC abrogated the hypersensitivity of Huh7 to cisplatin [33]. The FA pathway is implicated in the proton radiation sensitivity in non-small lung cancer cells [15]; in H460 cells, the depletion of FANCD2 with siRNA increased the RBE from 1.10 to 1.39. Thus, it is speculated that the increased RBE of Huh7 cells might be due to the defective FA pathway derived from genetic inactivation of FANCC.

Prolonged DNA damage responses were seen in proton-irradiated HCC cells (Fig 2B), reflecting more complex DSB formation by protons than X-rays. Cancer cells choose different DSB repair pathways between HR and NHEJ upon X-ray and proton irradiations, despite two radiations are low LET beams [14]. In lung and brain cancer cells, the inhibition of DNA-PKcs, an NHEJ pathway protein, rendered cells more sensitive to X-rays than protons and the depletion of RAD51, a HR pathway protein, showed the opposite effect, suggesting a relevant role of HR pathway in the proton-damaged DNA repair [14]. Our data suggested that NHEJ pathway may be important for proton-induced DNA damage repair in HCC cells, which is supported by previous reports showing targeting DNA-PKcs as an effective way for enhancing radiosensitivity to carbon ions [16–18]. Although BRCA1 silencing did not affect radiosensitivity in SNU449, we cannot rule out the possibility that the HR pathway may also contribute to the proton sensitivity in liver cancer.

Deregulation of DNA repair pathway genes including DNA-PKcs is frequently seen during HCC progression [34,35] and DNA-PKcs is considered a promising therapeutic target in HCC [21,22]. Pharmacological inhibition or gene silencing of DNA-PKcs enhances the radiosensitivity of liver cancer cells such as HepG2 [19], which is in accordance with our data using HepG2, SNU-449 and Hep3B cells. Proton radiosensitization by the depletion of DNA-PKcs in HCC cells may be caused by accumulating unrepairable DNA damage. In the DNA-PKcs-deficient HCC cells, proton irradiation induced more complex DNA damage, leading to the enhancement of cell percentage at the G2/M phase at 24 h (Fig 5A) and at the subG1 phase at 72 h (Fig 5B) of post-irradiation. The unrepairable DNA damages triggered the apoptotic cell death, which was the highest in the cells co-treated with DNA-PKcs siRNA and protons (Fig 6). The combination with DNA-PKcs siRNA and protons increased expression of pro-apoptotic genes such as Bak both transcriptionally and translationally. In contrast, the expression of anti-apoptotic genes such as Bcl-2 were only translationally regulated by the co-treatment. Increased ratio of Bak and Bcl-2 proteins represented a shift of the balance toward induction of apoptosis. SNU-449 cells are known to be resistant to other anticancer therapeutics including doxorubicin and sorafenib via upregulating pro-growth/survival signals [36–38]. Thus, targeting DNA-PKcs in combination with proton irradiation would be an effective way to treat such a chemo- and radio-resistant HCC.

It is now accepted that the biological effects of proton irradiation differ from those of X-rays, depending on genetic background. Our *in vitro* data indicate that proton RBE seems variable in liver cancer cell lines like other types of cancers. The RBE screen suggests that the functionality of DNA repair pathways including the NHEJ and FA pathways may impact on the proton sensitivity in liver cancer cells, as was previously seen in lung cancer cells [15]. Thus, this study supports the notion that there may be common genetic factors contributing to the RBE variations across cancer types. There are also RBE variations along proton beam path, particularly a sharp rise of RBE at the distal end of SOBP due to increased LET, which is an issue related to proton range uncertainty [39]. We evaluated RBE variations only at the mid of SOBP, so it would be intriguing to determine the effect of genetic variations on RBE at different positions of SOBP. Also, the use of animal models is needed for further validation. Together, a more comprehensive investigation of these RBE contributors will assist in moving toward precision medicine-based proton therapy.

## Conclusions

Proton beam therapy is being recognized as a reasonable treatment option for liver cancer due to superior physical properties. In this study, we found large variations in radiosensitivity and proton RBE among liver cancer cell lines as seen in other cancer types. Nonetheless, our findings do not encourage a clinician to use different RBEs from the standard assumption of 1.1 for protons. This study provides first evidence that genetic inactivation of DNA-PKcs, frequently deregulated in advanced liver cancer, enhanced sensitivity of liver cancer cells to proton beam therapy via induction of apoptotic death. Thus, targeting DNA-PKcs in combination with proton therapy could be a promising strategy for HCC treatment.

## Supporting information

S1 Fig Dose response curves of eight human liver cancer cell lines for X-rays and protons. For the clonogenic assay, each cell line was seeded and exposed to either X-rays or protons at 0, 2, 4, 6 and 8 Gy. After 1 to 2 weeks, cells were stained with crystal violet and colonies consisting of 50 or more cells were manually counted. Survival data were obtained from three independent experiments and were fitted with a linear quadratic model using an in-house program. Solid and dashed lines represent the best-fit curves for X-ray-irradiated and proton-irradiated cells, respectively.
(TIF)

S2 Fig Depletion of DNA-PKcs enhances proton-induced apoptosis in HepG2 cells. Apoptotic cell death was evaluated by using flow cytometry with annexin V and PI co-staining. Data represent mean ± SD. ***p < 0.001; n.s.: not significant.
(TIF)

S3 Fig Effect of DNA-PKcs depletion on the expression of pro-apoptotic/anti-apoptotic genes in SNU-449 cells. (**A**) Relative gene expression of DNA-PKcs in knockdown cells versus control siRNA cells. The gene expression was measured by using real-time qRT-PCR method as described in Materials and methods. (**B**) The effects of DNA-PKcs knockdown and X-ray/proton irradiation on the mRNA expression of Bak and Bcl-2. The mRNA expression was assessed by qRT-PCR. (**C**) The effects of DNA-PKcs knockdown and X-ray/proton irradiation on the protein expression of Bak and Bcl-2. The protein expression was assessed by western blot. The ratio of Bak/Bcl-2 was greatly increased by co-treatment with DNA-PKcs siRNA and proton irradiation.
(TIF)

S1 Dataset Data used to build graphs. (XLSX)
