# Supplementary figures and images for: Targeting DNA-dependent protein kinase sensitizes hepatocellular carcinoma cells to proton beam irradiation through apoptosis induction
Source: PLoS One. 2019 Jun 13;14(6):e0218049. doi: 10.1371/journal.pone.0218049 (PMC6563991; doi:10.1371/journal.pone.0218049)

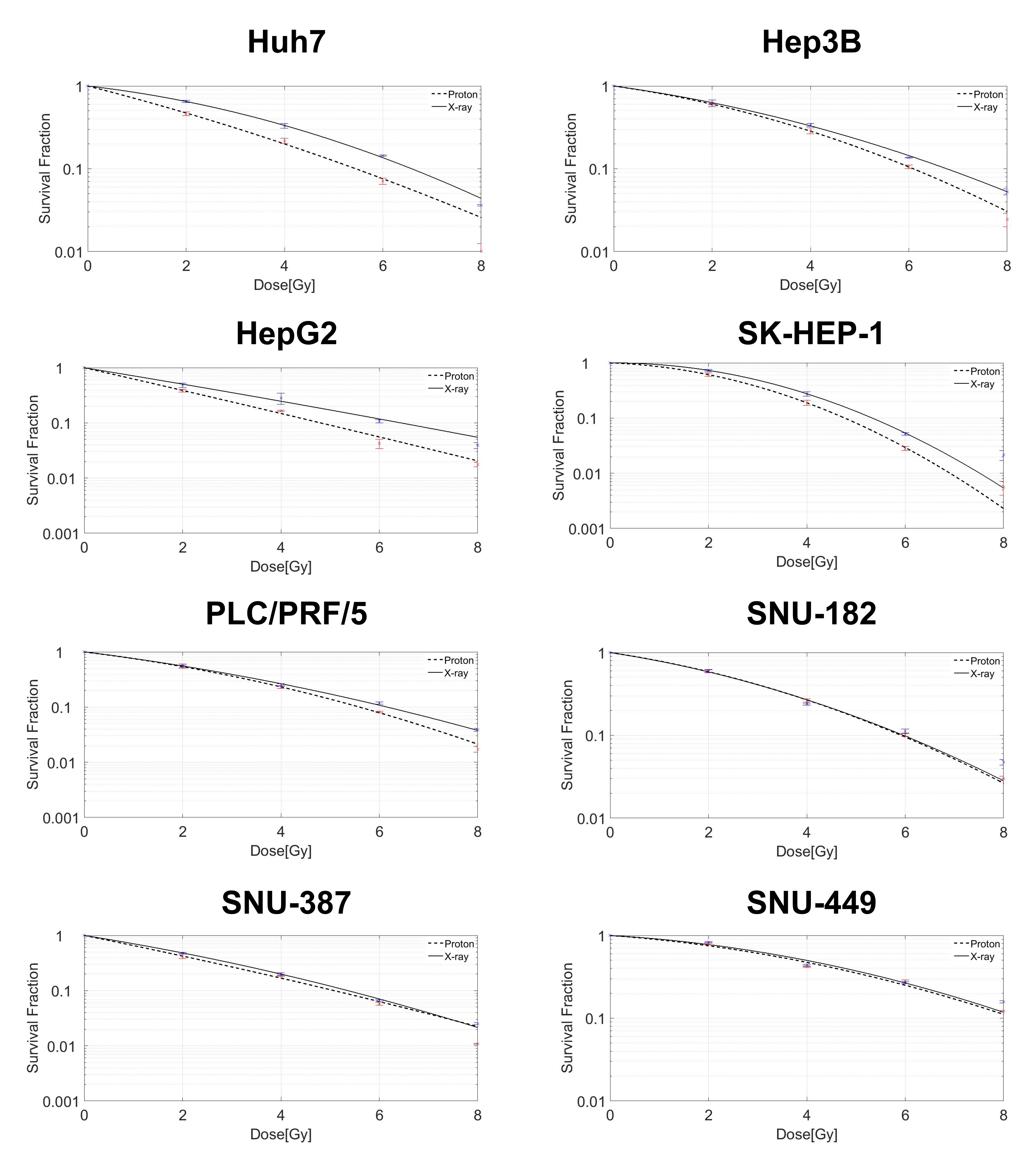

Supplement: S1 Fig — For the clonogenic assay, each cell line was seeded and exposed to either X-rays or protons at 0, 2, 4, 6 and 8 Gy. After 1 to 2 weeks, cells were stained with crystal violet and colonies consisting of 50 or more cells were manually counted. Survival data were obtained from three independent experiments and were fitted with a linear quadratic model using an in-house program. Solid and dashed lines represent the best-fit curves for X-ray-irradiated and proton-irradiated cells, respectively. (TIF) [file pone.0218049.s001.tif]

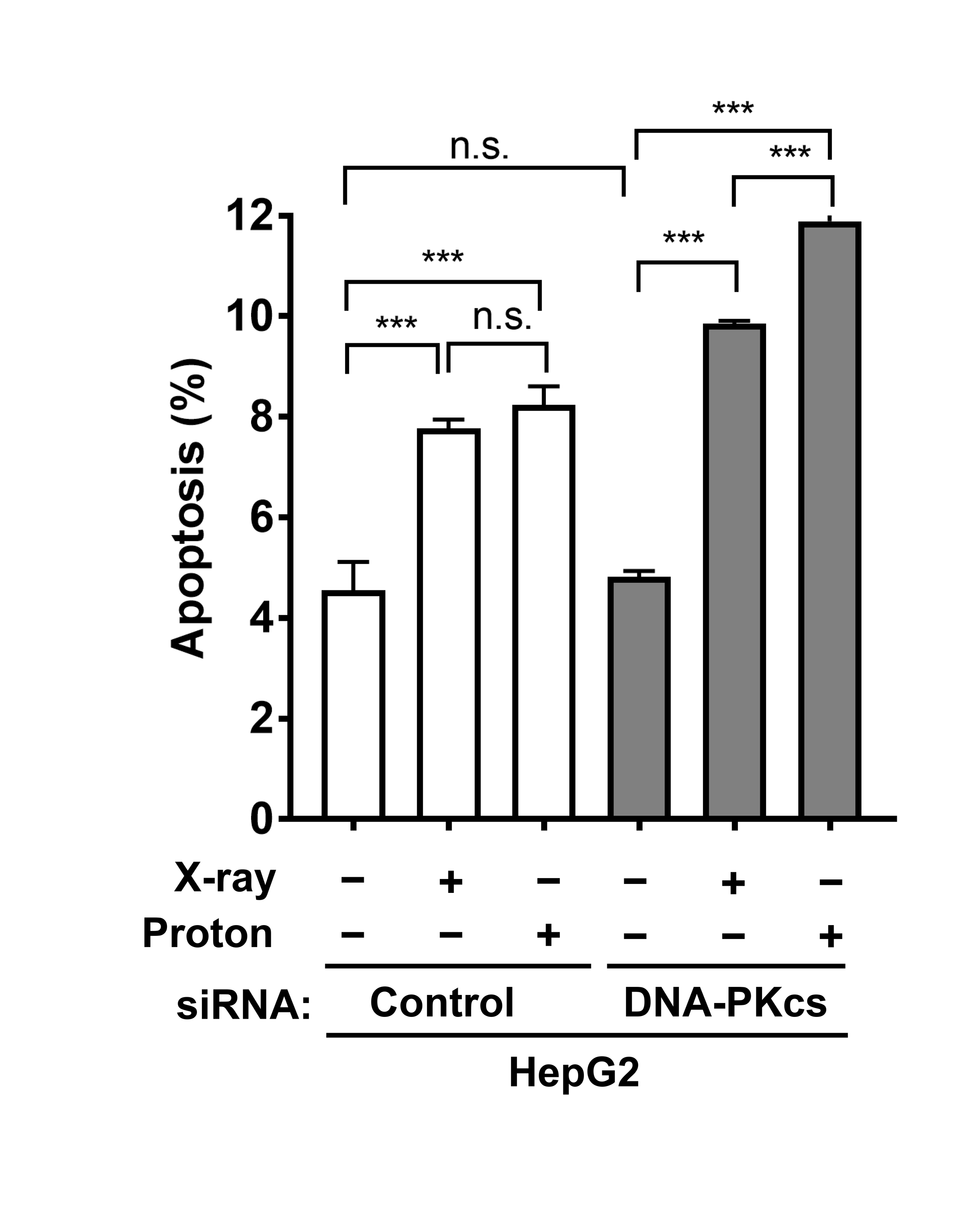

Supplement: S2 Fig — Apoptotic cell death was evaluated by using flow cytometry with annexin V and PI co-staining. Data represent mean ± SD. ***p < 0.001; n.s.: not significant. (TIF) [file pone.0218049.s002.tif]

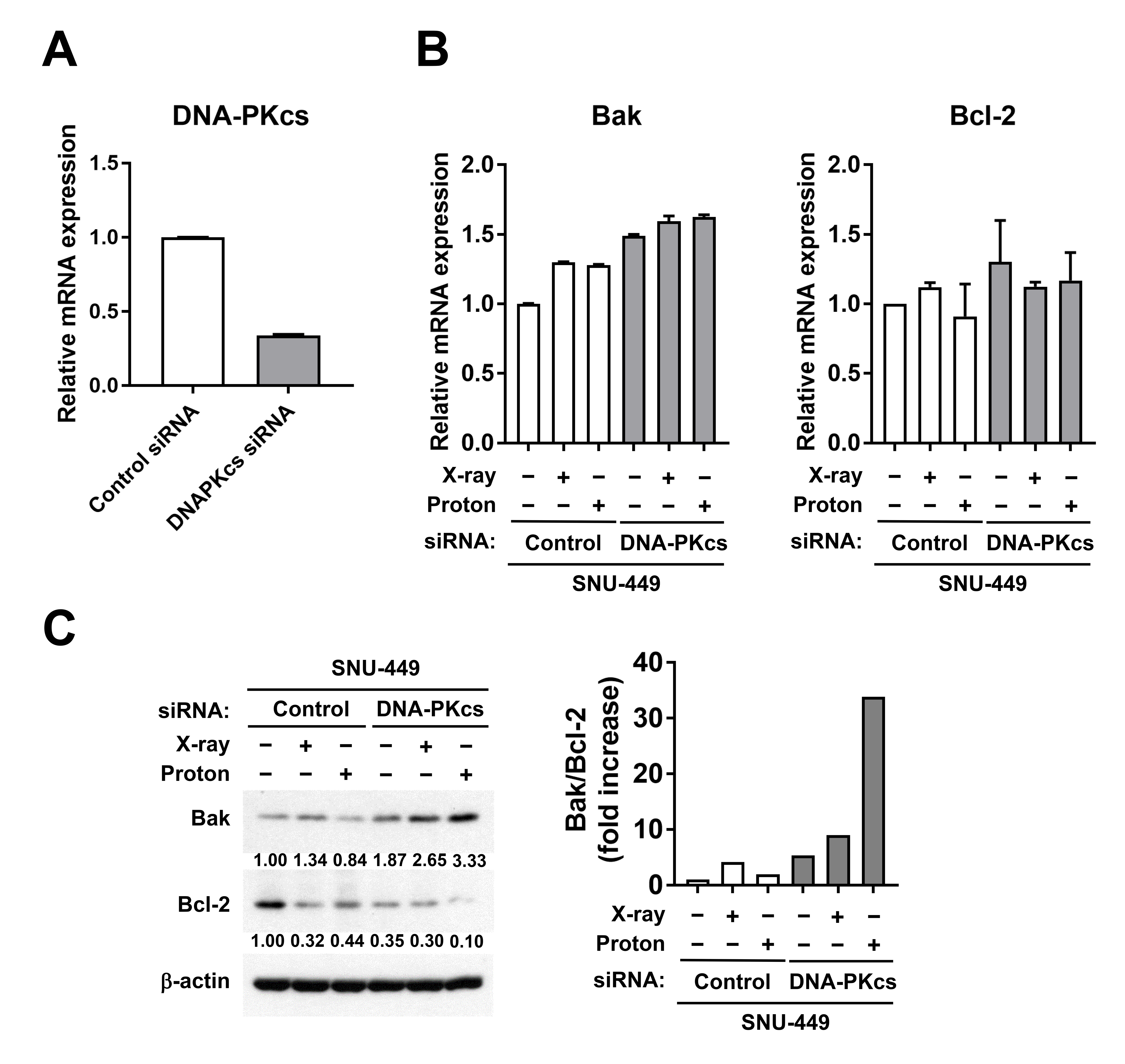

Supplement: S3 Fig — (A) Relative gene expression of DNA-PKcs in knockdown cells versus control siRNA cells. The gene expression was measured by using real-time qRT-PCR method as described in Materials and methods. (B) The effects of DNA-PKcs knockdown and X-ray/proton irradiation on the mRNA expression of Bak and Bcl-2. The mRNA expression was assessed by qRT-PCR. (C) The effects of DNA-PKcs knockdown and X-ray/proton irradiation on the protein expression of Bak and Bcl-2. The protein expression was assessed by western blot. The ratio of Bak/Bcl-2 was greatly increased by co-treatment with DNA-PKcs siRNA and proton irradiation. (TIF) [file pone.0218049.s003.tif]
